# Validation of the C-X-C chemokine receptor 3 (CXCR3) as a target for PET imaging of T cell activation

**DOI:** 10.1186/s13550-024-01142-1

**Published:** 2024-08-28

**Authors:** Sebastian Martin, Lennard Wendlinger, Béatrice Zitti, Mehdi Hicham, Viktoriia Postupalenko, Léo Marx, Greta Giordano-Attianese, Elisabetta Cribioli, Melita Irving, Alexandra Litvinenko, Radmila Faizova, David Viertl, Margret Schottelius

**Affiliations:** 1 Translational Radiopharmaceutical Sciences, Department of Nuclear Medicine, Department of Oncology, Centre Hospitalier Universitaire Vaudois (CHUV) and University of Lausanne (UNIL), Lausanne, 1011 Switzerland; 2 AGORA, Pôle de recherche sur le cancer, Lausanne, 1011 Switzerland; 3 SCCL Swiss Cancer Center Leman, Lausanne, 1011 Switzerland; 4 Debiopharm Research & Manufacturing SA, Campus “après-demain”, Rue du Levant 146, Martigny, 1920 Switzerland; 5 Department of Pathology and Immunology, University of Geneva, Geneva, Switzerland; 6 Ludwig Institute for Cancer Research, University of Lausanne, Epalinges, 1066 Switzerland; 7 Department of Oncology, University Hospital of Lausanne, Lausanne, 1011 Switzerland

**Keywords:** CXCR3, Immunotherapy, Chemokine receptor, T cell activation imaging

## Abstract

**Purpose:**

CXCR3 is expressed on activated T cells and plays a crucial role in T-cell recruitment to the tumor microenvironment (TME) during cell-based and immune checkpoint inhibitor (ICI) immunotherapy. This study utilized a ^64^Cu-labeled NOTA-α-CXCR3 antibody to assess CXCR3 expression in the TME and validate it as a potential T cell activation biomarker in vivo.

**Procedures:**

CXCR3^+^ cells infiltrating MC38 tumors (B57BL/6 mice, untreated and treated with *α*PD-1/*α*CTLA-4 ICI) were quantified using fluorescence microscopy and flow cytometry. A commercial anti-mouse CXCR3 antibody (α-CXCR3) was site-specifically conjugated with 2,2,2-(1,4,7-triazacyclononane-1,4,7-triyl)triacetic acid (NOTA) and radiolabeled with ^64^Cu. Saturation binding of [^64^Cu]Cu-NOTA-*α*-CXCR3 was investigated using CHO cells stably transfected with murine CXCR3. Biodistribution and PET imaging studies both at baseline and after 1 to 3 cycles of ICI, respectively, were carried out using different molar activities (10 GBq/µmol to 300 GBq/µmol) of [^64^Cu]Cu-NOTA-α-CXCR3.

**Results:**

Flow cytometry analysis at baseline confirmed the presence of CXCR3 + T-cells in MC38 tumors, which was significantly increased at day five after ICI (treated 33.8 *±* 17.4 vs. control 8.8 *±* 6.2 CD3^+^CXCR3^+^ cells/mg). These results were qualitatively and quantitatively confirmed by immunofluorescence of tumor cryoslices. In vivo PET imaging of MC38 tumor bearing mice before, during and after ICI using [^64^Cu]Cu-NOTA-α-CXCR3 (Kd = 3.3 nM) revealed a strong dependence of CXCR3-specificity of tracer accumulation in secondary lymphoid organs on molar activity. At 300 GBq/µmol (1.5 µg of antibody/mouse), a specific signal was observed in lymph nodes (6.33 ± 1.25 control vs. 3.95 ± 1.23%IA/g blocking) and the spleen (6.04 ± 1.02 control vs. 3.84 ± 0.79%IA/g blocking) at 48 h p.i. Spleen-to-liver ratios indicated a time dependent systemic immune response showing a steady increase from 1.08 *±* 0.19 (untreated control) to 1.54 *±* 0.14 (three ICI cycles).

**Conclusions:**

This study demonstrates the feasibility of in vivo imaging of CXCR3 upregulation under immunotherapy using antibodies. However, high molar activities and low antibody doses are essential for sensitive detection in lymph nodes and spleen. Detecting therapy-induced changes in CXCR3^+^ T cell numbers in tumors was challenging due to secondary antibody-related effects. Nonetheless, CXCR3 remains a promising target for imaging T cell activation, with anticipated improvements in sensitivity using alternative tracers with high affinities and favorable pharmacokinetics.

**Supplementary Information:**

The online version contains supplementary material available at 10.1186/s13550-024-01142-1.

## Introduction

Generally, the success and therapeutic efficacy of immunotherapies intrinsically rely on the presence and activity of T cells in the TME. Both for ICI as well as cell-based immunotherapies (adoptive tumor infiltrating lymphocyte (TIL) and chimeric antigen receptor T cell (CART cell)), no or negligible anti-tumor responses are observed, when CD8^+^ effector and CD4^+^ helper T cells are sparse or absent from the TME or if they are inactive due to immunosuppressive signaling in and by the tumor and TME [1–3]. Unfortunately, tumor biopsies can only provide spatial and temporal spotlight information on the composition of the TME in a given cancer lesion and do not capture the dynamics of the immune cell infiltrate over time and in response to therapy. In contrast, nuclear imaging methods such as positron emission tomography (PET) and single photon emission computed tomography (SPECT) allow the longitudinal non-invasive whole-body visualization of distinct cell populations. Fueled by the development of dedicated, highly specific radiopharmaceuticals in recent years, the non-invasive quantification of tumor infiltration with distinct immune cell subsets, e.g. CD8^+^ cytotoxic T cells [4], is now rapidly entering clinical practice, providing crucial information for patient selection and on early response to immune checkpoint blockade.

Furthermore, ongoing efforts are directed towards not only visualizing the presence, quantity and localization of T cells in the TME, but on imaging their activation state, which may represent a more robust predictor for the success of immunotherapies than T cell presence alone [5]. Various molecular targets have been investigated as biomarkers for T cell activation in recent years. These include OX40 [6], CD69 [7], inducible T cell costimulator (ICOS) [8, 9], interleukin-2 receptor (IL-2R) [10, 11], interferon gamma (INF-*γ*) [12], Granzyme B [13], and T-cell metabolism [14]. In preclinical PET imaging studies using specifically designed targeted tracers, ranging from full-sized antibodies (OX40, CD69, ICOS, INF-*γ*) over proteins (IL-2) and peptides (Granzyme B) to small molecules (metabolism), all these targets were found to be relevant molecular markers for T-cell activity, with the accumulation of the respective targeted tracer in the tumor or site of inflammation (pre and/or post-therapy) closely correlating with the presence of activated T cells in the tissue of interest and with therapy response [6–14]. Of these, both the metabolic tracer [^18^F]F-AraG (NCT04726215, NCT05096234) as well as the human Granzyme B targeted [^68^Ga]Ga-NOTA-human-GZP (NCT04169321) have recently been translated into clinical studies.

Another highly interesting cell surface marker associated with Tell activation is the CXCR3 [15]. CXCR3 directs the temporal and spatial migration of activated T cells and natural killer cells towards sites of high expression of its native ligands, the INF-*γ* inducible proteins, C-X-C chemokine ligands 9 (CXCL9), CXCL10, and CXCL11 [16, 17] creating an inflammatory environment. Both in the context of ICI and of TIL-therapies, the CXCR3-chemokine axis was identified as a key player in the efficient recruitment of T cells to the TME as well as their expansion in the TME and their antitumor-activity [15, 18, 19]. Thus, CXCR3 represent a highly promising target for nuclear imaging, possibly allowing the visualization of T-cell distribution, homing and activation in the immunotherapy setting, both as a pretherapeutic prognostic marker as well as for assessing response to treatment. To date, only two reports on CXCR3-targeted nuclear imaging have been published, both of which use inflammation models (allograft rejection, atherosclerosis) for tracer evaluation [20, 21]. However, both radiopharmaceuticals investigated, [^125^I]CXCL10 [20] and an ^18^F-labeled small molecule CXCR3 inhibitor [21] accumulated specifically in inflammatory regions infiltrated with CXCR3-expressing T cells, and the respective signal intensity correlated with CXCR3^+^ T cell density.

Based on these encouraging findings, we designed the present study to validate CXCR3 as an imaging target in the TME and as a potential biomarker for therapy control and the prediction of therapy outcome in the immunotherapy setting. For the initial quantification of target expression at baseline and after several cycles of immunotherapy (anti-programmed cell death protein 1 (αPD-1)/anti-cytotoxic T-lymphocyte-associated protein 4 (αCTLA-4)) in a MC38 syngeneic colon cancer model via CXCR3-targeted PET, we used a commercially available anti-mouse CXCR3 antibody (α-CXCR3). The monoclonal antibody (mAb) was site-specifically conjugated with a 2,2,2-(1,4,7-triazacyclononane-1,4,7-triyl)triacetic acid (NOTA) chelator using the AbYlink™ technology and radiolabeled with ^64^Cu.

## Methods

### Antibody conjugation and characterization

Upon buffer exchange using an Amicon 50 kDa centrifugal filter, a commercially available anti-mouse CXCR3 mAb (clone: CXCR3-173, #Cat: BE0249, BioXcell) was site-specifically conjugated with NOTA according to a previously published protocol conjugated with (AbYlink^™^, Debiopharm) [22]. Briefly, the antibody (1 eq.) was resuspended in carbonate buffer (0.2 M, pH = 9). The conjugation mix (2.2 eq.) containing AbYlink-Bn-NOTA was added and the reaction was gently agitated for 2 h at RT. Next, the reaction mixture was quenched for 5 min at RT with glycine buffer (0.4 M, pH 2.5, 50 µL/mg antibody). Purification was performed using a EMP Biotech CentriPure PF10 centrifugal device. The product was eluted using glycine buffer (0.1 M, pH = 2.5) into a tube containing the neutralization buffer (0.22 mL/mL of eluate, 0.5 M phosphate buffer, pH = 8.5). The average degree of conjugation (DoC) was determined by mass spectrometry analysis operating in protein mode (Sion, EPFL) and the final antibody concentrations were determined by a NanoDrop One/One spectrophotometer (Thermo Fisher).

### Radiolabeling

A solution of ^64^Cu dichloride in 0.1 N HCl was obtained from ARRONAX (Saint Herblain, France). To a defined volume of the ^64^CuCl_2_ solution supplied by the manufacturer, 1/10 (v/v) of metal free sodium acetate 2.5 M (pH = 8.5) was added. Then, the radiolabeling precursor (30–80 µg) in acetate buffer (0.1 M, pH = 5.5) was added. After 30 min incubation at 40 °C, 1 mM ethylenediaminetetraacetic acid (EDTA) (pH = 7.0, Sigma Aldrich, St. Quentin Fallavier, France) was added to obtain a final concentration of 0.01 mM to complex free ^64^Cu^2+^. Molar activity (MA) was calculated by the decay-corrected activity from the synthesis start divided by the molar mass of a full-size antibody (150 kDa).

### Size exclusion chromatography

Size exclusion chromatography (SEC) was performed on a Shimadzu (LC20AT, SPD-M20A) system using a XBridge protein BEH 200 A Sect. 3.5 μm, 7.8 × 300 mm (Waters, Baden-Dättwil, Switzerland) size exclusion column and phosphate buffer (0.1 M, pH 6.8) containing 342 mM NaCl at a constant flow of 1 mL/min as mobile phase. UV detection was performed at 220, 254 and 280 nm, and the radioactivity signal was detected using a GABI well-type scintillation detector (Elisa-Raytest). Unconjugated α-CXCR3: R_t_ (retention time) 8.5 min, purity 99.2%; NOTA-α-CXCR3: R_t_ 8.5 min, purity 98.8%; [^64^Cu]Cu-NOTA-α-Cxcr3: R_t_ 8.6 min, radiochemical purity (RCP) > 99%.

### Stability in human serum

To a volume of 500 µL of human serum, 50 µL of radiotracer were added. The mixture was incubated at 37 °C. SEC analyses were carried out after 24 and 48 h of incubation. Before injection, 100 µL of the sample were passed through a 0.2 μm filter.

[^64^Cu]Cu-NOTA-α- CXRCR3: R_t_ = 8.6 min, RCP after 24 and 48 h > 95%.

### Instant thin layer chromatography (iTLC)

In-process reaction controls were performed using iTLC. The radiochemical yield (RCY) of [^64^Cu]Cu-NOTA-α-CXCR3 was determined by instant thin layer chromatography (iTLC) using dried iTLC-SG Glass microfiber chromatography paper impregnated with silica gel (Agilent Technologies, Folsom, CA 95630) as stationary phase and citrate buffer (0.1 M, pH = 4.5) as eluent. For iTLC evaluation, a Scan-RAM radio-TLC scanner (LabLogic) and Laura software (LabLogic, Version 6.0.3) were used.

[^64^Cu]Cu-EDTA, R_f_ = 1; [^64^Cu]Cu-NOTA-α-CXCR3, R_f_ = 0.

### Saturation binding assay

Transfected CHO-CXCR3 cells were maintained and expanded using Ham’s F-12 Nutrient Mix cell culture media (Thermo Fisher Scientific) supplemented with 10% fetal bovine serum (FBS) and 1% penicillin/streptomycin in a cell incubator at 37 °C, 5% CO_2_. 2 × 10^6^ CHO-CXCR3 cells were seeded in in a 6-well plate one day before the experiment. On the day of the experiment, the growth medium was removed and the cells were washed once with cold assay buffer (0.5% BSA in PBS). Then, 800 µL of assay buffer were added into each well, and the radiolabeled antibody (100 µL, increasing concentrations, *n* = 3 per concentration) was added. Additionally, 100 µL assay buffer were added to the wells to reach a final volume of 1 mL. Final concentrations of [^64^Cu]Cu-NOTA-α-CXCR3 were as follows: 25, 10, 1, 0.5, 0.25, 0.125 and 0.063 nM (*n* = 3 per radioligand concentration). To determine non-specific binding at each radioligand concentration, radioligand binding was also investigated in the presence of a 100-fold excess of unlabeled α-CXCR3. Here, 100 µL of α-CXCR3 antibody in assay buffer was added to the well to reach a final volume of 1 mL (*n* = 3 per radioligand concentration). Plates were incubated for 2 h at 4 ^°^C. The supernatant was removed and combined with 1 mL of assay buffer using for washing the cells. The cells were then lysed with 1 M NaOH. The cell lysate was then combined with 1 mL of assay medium using for washing the wells. Both the activities in the supernatants and the respective lysates were quantified using a y-counter (Wizzard 3, Perkin Elmer). Non-specific binding and total binding were analyzed by GraphPad prism version 9.1.0.

### Animal experiments

Animal experiments were conducted according to the protocols approved by the Veterinary Authorities of the Canton Vaud (license VD 3781) and in accordance with the Swiss Animal Welfare Act. Six to eight week-old female Bl57/6 mice were purchased from Charles River Laboratories (France, L’Arbresle). MC38 cells were maintained in RPMI 1640 GlutaMAX^™^ cell culture media (Thermo Fisher Scientific) supplemented with 10% FBS and 1% penicillin/streptomycin in a cell incubator at 37 °C, 5% CO_2_. Tumor engraftment and the subsequent treatment schedule were conducted as described previously [23]. Briefly, MC38 tumors were engrafted on the right flank by subcutaneous injection of 2 × 10^6^ cells in 100 uL PBS. The first treatment with immune checkpoint inhibitors (ICIs) was initiated at day 7 post-engraftment. The checkpoint inhibitor combination consisted of 200 µg αCTLA-4 (clone: 9D9, #Cat: BP0164, BioXCell) and 200 µg αPD-1 (clone: 29 F.1A12, #Cat: BE0273, BioXCell) and was injected i.p. in a total volume of 100 µL of sterile PBS. Additional treatments were administered on day 10 and on day 13 after tumor inoculation. At day 10 (untreated and 1xICI treated) and day 12 (2xICI treated) post-inoculation, MC38 tumors were dissected for flow cytometry, immunofluorescence, or immunohistochemistry analysis. Tumor size was monitored using caliper measurements and by applying the following formula to calculate the tumor volume: *tumor volume* = (*length/*2)* width^2^.

### Flow cytometry

After harvesting the MC38 tumors, single cell suspensions were immediately generated by cutting the tumor in small pieces and incubating the tissue in 1 mL FBS containing collagenase IV (0.2 mg/L) and DNAse I (2 µg/L). Upon incubation at 37 ^°^C for 20 min, the tissue was passed through a cell strainer and the cell suspension was washed two times with cold PBS. The cells were stained at room temperature for 25 min in flow cytometry buffer (PBS suppl. with 1 mM EDTA, 1% FBS). Control and FMO stains were performed on splenocytes. The following antibodies were utilized: Fc block (clone: 93, #Cat: 101302, Biolegend), DAPI (Invitrogen), CD45 (clone: 30-F11, #Cat: 103132, Biolegend), CD3 (clone: 145-2c11, #Cat: 100306, Biologend), CD4 (clone: Gk-1.5, #Cat: 552051, Biolegend), CD8: (clone 53 − 6.7, #Cat: 100712, Biolegend), CXCR3 (clone: CXCR3-173, #Cat: 126502, Biolegend). The antibody concentrations were 1 µg/mL in flow cytometry buffer. DAPI was added to each sample to yield a final concentration of 0.5 µg/mL and the cells were washed once. The cells were fixed with 2% PFA in PBS on ice. Precision count beads (#Cat: 424902, Biolegend) were added to each tumor sample and compensation beads were used (#Cat: 01-3333-42, Thermo Scientific) to set up the panel. The analysis was performed on a BD FACSymphony A5 Flow Cytometer on the same day. The data was evaluated using FlowJo V10.7.1.

### Immunofluorescence

Immunofluorescence staining was performed using acetone fixed tissue slices. The tissues were blocked with donkey and goat serum (10%) for 20 min. After washing the samples in 0.3% triton in PBS, tissues were incubated with the primary antibodies (1:50, diluent DAKO S3022) for 60 min at RT. After another washing step, the cells were incubated with the respective fluorescent secondary antibody (1:500, diluent DAKO S3022) for 30 min at room temperature. DAPI (Sigma, #Cat: D9542, 1:3000 dilution in PBS) was used to stain the cell nuclei. After the slices had been rinsed with distilled water, the slices were covered with water-based mounting media (DAKO S3023) and glass square coverslips. Negative controls were incubated with the fluorescent secondary antibodies only. For fluorescence microscopy image evaluation, the QuPath version 0.3.2 software for digital pathology image analysis was used by applying a trained object classifier for the positive cell annotation [24].

### Immunohistochemistry

Immunohistochemistry was performed on acetone fixed slices. The tissues were blocked with goat serum (10%) for 20 min. After washing the samples in 0.3% triton in PBS, tissues were incubated with the respective primary antibodies (1:200, diluent DAKO S2022) at room temperature for 40 min. After another washing step, the slices were incubated with the secondary antibody (HRP-conjugated anti-rabbit, DAKO K4003) for 30 min at room temperature. After another washing step, the staining was completed by DAB chromogen revelation (DAKO K3468). Harris Gill II was applied as control color. Subsequently, the slides were dehydrated in xylol and covered with permanent mounting. Negative tissues were stained with the secondary antibody (HRP anti-rabbit) only.

### Autoradiography

Autoradiography experiments were performed on a BeaQuant S real time autoradiography instrument (ai4R, Nantes). The radiotracer [^64^Cu]Cu-NOTA-α-CXCR3 (2–3 MBq, 3 µg, containing 50 µg polyclonal Armenian hamster IgG (BioXCell, #BE0091)) was injected, and at 24 h p.i., the spleens were harvested and transferred to optimal cutting temperature compound (O.C.T. Tissue-Tek, Sakura Finetek). Spleens were immediately frozen on dry ice. Slices (8 μm) were cut and left to dry on the glass plate for 2 h at RT. The autoradiography measurement was performed overnight for 24 h. Images were analyzed using BEAMAGE (v3.3.X) software.

### Biodistribution study

ICI treated MC38 tumor bearing mice were injected intravenously into the tail vein with 1.5 µg (3 MBq) or 15 µg (1 MBq) [^64^Cu]Cu-NOTA-α-CXCR3 in 100 µL sterile 0.9% NaCl on day 9 (untreated and 1xICI treated) and on day 12 (2xICI treated) post-inoculation. To reduce Fc-mediated non-specific tracer accumulation, mice were coinjected with 50 µg polyclonal Armenian hamster IgG (BioXCell, #BE0091). To quantify CXCR3-specific tracer accumulation, an additional group of mice (*n* = 3–5) was coinjected with a 100-fold excess of unconjugated α-CXCR3. The animals were sacrificed 24 h (15 µg, 1 MBq injected activity) or 48 h (1.5 µg, 3 MBq injected activity) post-injection of the ^64^Cu-labeled antibody, and the organs of interest were dissected. The activity in the weighed tissue samples was quantified using a Wizard 3 y-counter (Perkin Elmer, Schwerzenbach). Data are expressed in %IA/g tissue (mean *±* SD) and are corrected for residual injection-related activity in the tail.

### PET/CT imaging

PET/CT images were acquired at 24 h p.i. on an Albira PET/SPECT/CT scanner (Bruker Biospin Corporation, Woodbridge, CT, USA) Mice were injected intravenously with [^64^Cu]Cu-NOTA-α-CXCR3 (3 MBq, 1.5 µg) in saline. As described for the biodistribution study, CXCR3-specificity of tracer accumulation was determined by coinjection of a 100-fold excess of unlabeled α-CXCR3 mAb. During image acquisition (static scan, 20 min, 32 × 32 0.5 mm, followed by a 10 min CT scan), the animals were anesthetized using isoflurane (1.5% alveolar concentration). During imaging, the body temperature and respiration rate was constantly monitored. Image reconstruction was performed by using Albira reconstructor (version NMI3.3), and the images were analyzed using PMOD software (V6.3.4, Bruker).

### Statistical analysis

All data were evaluated using GraphPad Prism version 9.1.0 for Windows, GraphPad Software, San Diego, California USA. A two-way ANOVA test followed by a Tukey’s multiple comparisons test was applied to determine the significance (*P* < 0.05) of the differences in CXCR3 positivity between the respective flow cytometry sample groups. A two-way ANOVA test followed by a Dunnett’s multiple comparisons test was applied to determine the significance (*P* < 0.05) in the differences in tracer uptake between groups in the biodistribution experiments. A one-way ANOVA test followed by a Dunnett’s multiple comparisons test was equally applied to determine the significance (*P* < 0.05) of the differences in the organ/liver or organ/blood ratios.

## Results

### Confirmation of therapy dependent infiltration of MC38 tumors with CXCR3+ cells

**Fig. 1:**
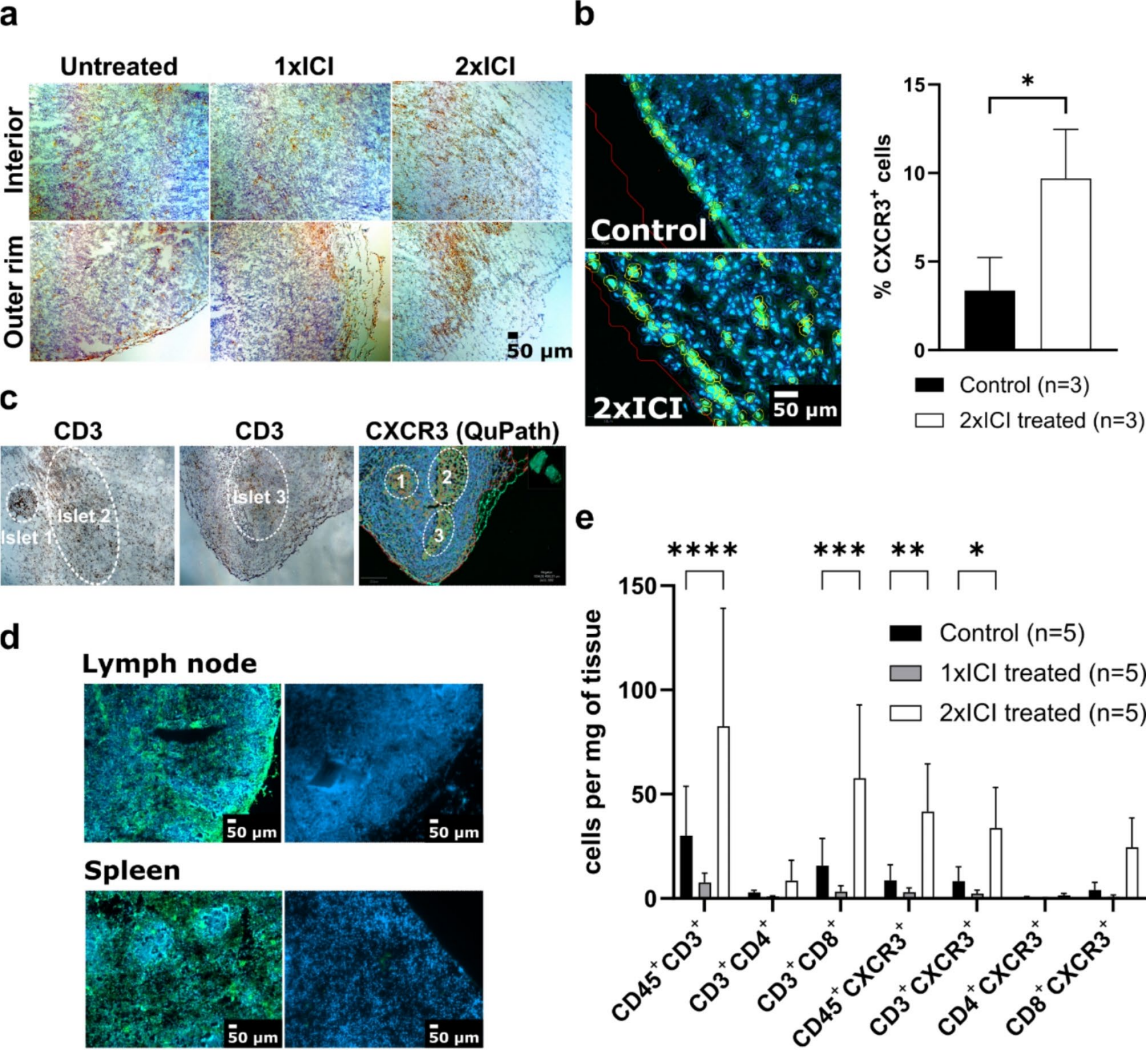
(**a**) CD3 immunohistochemistry of MC38 tumors treated with either one (1xICI) or 2 cycles of αPD-1/αCTLA-4 ICI (2xICI); (**b**) Fluorescence microscopy of MC38 tumor slices. Cells (DAPI in blue, CXCR3 in green) were annotated and counted using QuPath; (**c**) Overlap regions of MC38 cryoslices from immunohistochemistry (CD3) and immunofluorescence (CXCR3) images; (**d**) Baseline CXCR3 immunofluorescence in untreated lymphoid tissues (left), with negative controls (stained without primary antibody) on the right; (**e**) Flow cytometry analysis of MC38 tumors treated either with one or two cycles of ICI

To confirm and quantify the infiltration of subcutaneous MC38 syngeneic colon cancer tumors with CXCR3-expressing T cells both at baseline and after ICI therapy (α-PD-1/α-CTLA-4 combination treatment) in a sufficient number for imaging, the following cohorts were investigated using imunohistochemistry (IHC) and immunofluorescence (IF) methods: [1] an untreated control group [2], a treated group receiving one cycle of ICI therapy and [3] a treated group receiving two cycles of ICI therapy at days 7 and 10 post tumor inoculation, respectively. IHC of untreated tumors and tumors treated with only one dose of ICI showed weak CD3^+^ cell infiltration that was restricted to the outer rim of the tumor (Fig. 1A). In contrast, after two therapy cycles, the tumors showed an increased and more homogeneous infiltration with CD3^+^ T cells. Immunofluorescence revealed the presence of CXCR3-expressing T cells both in untreated and in treated tumors as well as in the control tissues spleen and lymph nodes. The corresponding annotated tissue slides are shown in Fig. 1A-D. While only 3.4 ± 1.9% CXCR3^+^ cells were detected in the untreated MC38 tumor tissues, two cycles of therapy cycles led to a significant increase in CXCR3^+^ cells (9.7 ± 2.8%). This observation is in accordance with the findings in CD3^+^ IHC. Again, treated tumors show increased and more homogenous infiltration than untreated tumors, where CXCR3^+^ cells are mainly localized on the outer rim of the tumor. Furthermore, more CXCR3^+^ cell clusters were detected in the interior of the tumors which received two treatment cycles (Fig. 1C).

To further corroborate the above data from the tissue analysis via IHC and IF, we additionally performed an in-depth analysis of the composition of the TME by flow cytometry, with lymphocytes being gated using CD45 as global immune cell marker. The tumors of the cohort which had received two therapy cycles showed a significant increase in CD3^+^ T cells as well as in the CD8^+^ T cell fraction (see Fig. 1E). This cohort also exhibited a global increase in CXCR3^+^ lymphocytes (8.7 *±* 6.6 vs. 41.7 ± 20.5 CXCR3^+^ cells/mg). This and a significant increase in CD3^+^CXCR3^+^ T cell numbers (8.8 *±* 6.2 vs. 33.8 *±* 17.4 CD3^+^CXCR3^+^ cells/mg) confirmed the observation from the IF analysis. After one treatment cycle, however, no changes in immune cell infiltration were observed three days after therapy. As demonstrated, T cell infiltration in the MC38 tumors was robust and up to threefold enhanced after ICI therapy, and therefore the chosen MC38 tumor model seemed appropriate for subsequent imaging studies.

### Antibody conjugation and radiolabeling

**Fig. 2:**
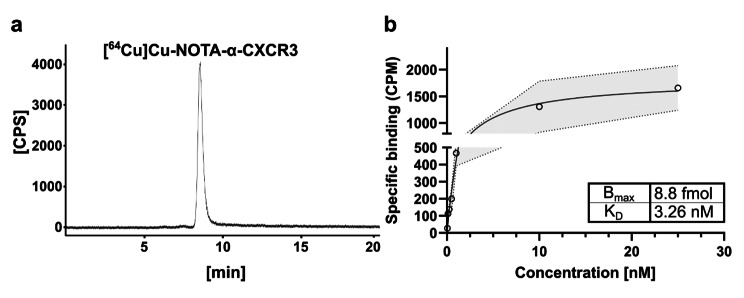
(**a**) Radio-SEC chromatogram of [^64^Cu]Cu-NOTA-α-CXCR3 (R_t_ = 8.6 min, RCP > 99%); (**b**) Saturation binding of [^64^Cu]Cu-NOTA-α-CXCR3 to murine CXCR3 (stably transfected CHO cells)

The Fc-specific conjugation of the commercially available α-CXCR3 mAb with the NOTA chelator using the AbYlink^™^ technology (see Suppl. Figure S1) [22] proceeded efficiently, yielding NOTA-α-CXCR3 in 98.8% purity and with a final average degree of conjugation (DoC) of 1.5, as determined by mass spectrometry (Suppl. Figure S2). Of note, NOTA-α-CXCR3 was found to remain fully stable for > 12 months at 4 ^°^C in labeling buffer (NaOAc, 0.1 M, pH = 5.5). Since high molar activities (MAs) are indispensable for sensitive target detection at comparably low expression levels, the same amount of NOTA-α-CXCR3 (31.3 µg, 0.2 nmol) was labeled with increasing ^64^Cu activities (10 to 50 MBq). The maximum achievable MA for [^64^Cu]Cu-NOTA-α-CXCR3 under the used standard labeling conditions was 690 GBq/µmol, with a radiochemical yield (RCY) of 96.8% (Suppl. Figure S3). For preparations with lower MA, RCYs of > 99% were consistently obtained (see Fig. 2a).

### In vitro characterization of [^64^Cu]Cu-NOTA-α-CXCR3

The binding affinity (K_D_) of the radiolabeled mAb was determined by a saturation binding assay using CHO cells stably transfected with CXCR3 (see Fig. 2b and Suppl. Figure S4). The antibody showed specific binding to the transfected cells with a K_D_ of 3.26 nM and a B_max_ of 8.8 fmol CXCR3 receptors per 10^6^ cells (5280 receptors per cell). Additionally, [^64^Cu]Cu-NOTA-α-CXCR3 was found to be stable in human serum for 48 h at 37 ^°^C (Suppl. Figure S5).

### In vivo characterization of [^64^Cu]Cu-NOTA-α-CXCR3 in tumor-free mice

**Fig. 3:**
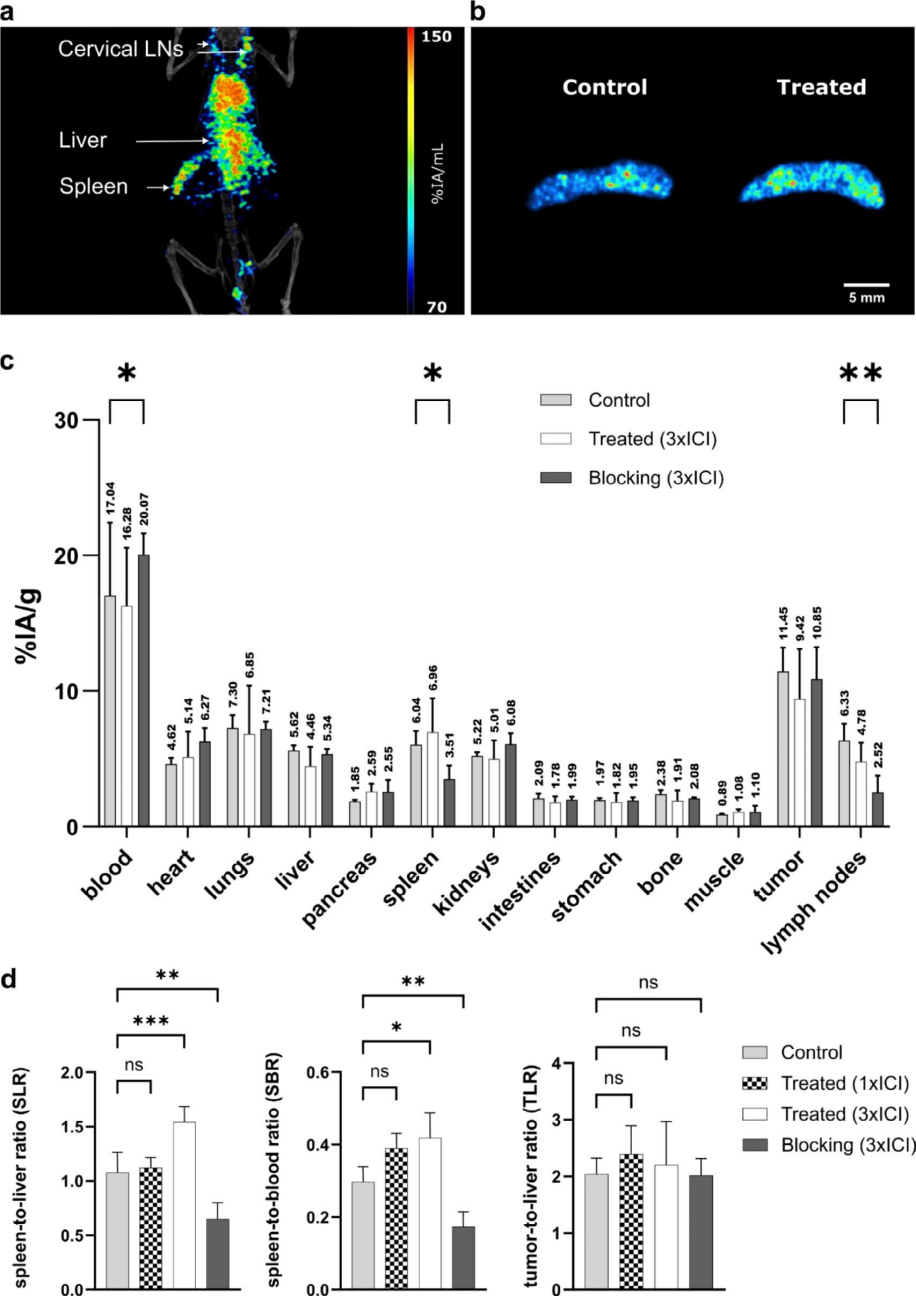
(**a**) PET/CT of a healthy C57Bl/6 mouse 48 p.i. of [^64^Cu]Cu-NOTA-α-CXCR3 (**b**) Autoradiography of spleens of healthy mice, either untreated (control, *n* = 1) or treated with 2 cycles of ICI (2xICI, *n* = 1)); (**c**) Biodistribution of [^64^Cu]Cu-NOTA-α-CXCR3 in MC38 tumor bearing C57Bl/6 mice at 48 h p.i. Uptake values are expressed as %IA/g (%-injected activity per gram of tissue) and are means ± SD (*n* = 5 mice per group). Groups include untreated mice (Control), mice treated with 3 cycles of ICI (Treated 3xICI). Blocking of 3xICI treated was conducted using a 100-fold molar excess of α-CXCR3 antibody. A two-way ANOVA was applied to confirm the statistcial significance (*P* < 0.05) of the differences in tracer uptake compared to the control cohort. (**d**) Spleen-to-liver, spleen-to-blood and tumor-to-liver ratios for the different cohorts as determined via the biodistribution study (Suppl. Table S1)

To evaluate the general biodistribution of the ^64^Cu-labeled antibody in vivo, orienting PET/CT imaging was performed 48 h p.i. in a tumor-free WT mouse (see Fig. 3a). This preliminary image demonstrated a potentially CXCR3-mediated radiotracer accumulation in the spleen and cervical lymph nodes as well as excretion-related uptake in the liver. To investigate whether a systemic activation induced by ICI treatment could be detected in healthy mice, an initial autoradiography experiment (see Fig. 3b) was performed. Spleens from an untreated control and an ICI treated mouse were dissected and snap-frozen, and tissue slices were prepared. Quantitative real-time autoradiography revealed a significant increase in activity, from 0.36 ± 0.002 (control) to 0.45 ± 0.01 (2xICI) counts per second (cp/s), was observed, hinting towards an increase in CXCR3-expression in the spleen due to systemic T-cell activation by ICI treatment. However, to prove CXCR3-specificity of [^64^Cu]Cu-NOTA-α-CXCR3 uptake in lymphoid tissues, detailed biodistribution and imaging studies were performed in MC38 tumor bearing mice.

### Biodistribution of [^64^Cu]Cu-NOTA-α-CXCR3 in MC38 bearing mice

The biodistribution of [^64^Cu]Cu-NOTA-α-CXCR3 in C57BL/6 mice bearing subcutaneous MC38 tumors at 24 h p.i. was first investigated using an antibody dose of 15 µg (MA = 10 GBq/µmol) per animal (Suppl. Figure S6). To evaluate CXCR3-specificity of tracer binding to secondary lymphoid organs (lymph nodes, spleen), mice were either injected with tracer only (control group) or coinjected with a 100-fold molar excess of unlabeled antibody (blocking). Under these conditions, no CXCR3-specific tracer accumulation was observed in the target organs. Since target expression was assumed to be comparably low at baseline (no treatment), the presence of relatively high amounts of unlabeled antibody in the low-molar-activity-preparation of [^64^Cu]Cu-NOTA-α-CXCR3 was thought to be responsible for already blocking all available binding sites, even in the control group. Thus, and due to the relatively high blood concentration of [^64^Cu]Cu-NOTA-α-CXCR3 at 24 h post-injection (p.i.), a second set of biodistribution studies was performed at 48 h p.i. and at a ten-fold lower antibody dose (1.5 µg, MA = 300 GBq/µmol) (Fig. 3c). Under these conditions, specific binding of [^64^Cu]Cu-NOTA-α-CXCR3 to CXCR3-expressing cells in spleen (*P* = 0.038) and lymph nodes (*P* = 0.002) was successfully confirmed by a blocking experiment (see Fig. 3c and d). Unexpectedly, [^64^Cu]Cu-NOTA-α-CXCR3 accumulation in the MC38 tumors at baseline was found to be high, but not CXCR3-specific (not blockable). Of note, due to the target saturation by unlabeled α-CXCR3, the amount of circulating radiotracer was higher in the blood pool of the blocking cohort compared to the control group (*P* = 0.011).

To investigate the in vivo effects of ICI therapy on CXCR3 expression in the TME as well as systemic effects, two therapy cohorts receiving either one (1xICI) or three (3xICI) cycles of checkpoint inhibitor treatment (α-PD1/α-CTLA-4) were included (for treatment schedule see Figure S7). A comprehensive table including the biodistribution data for all investigated cohorts is provided in the supplementary information (see Suppl. Table S1). Previous experiments in our lab using flow cytometry and IF had revealed that therapy response in the TME can be visualized earliest at five days post-treatment. Thus, [^64^Cu]Cu-NOTA-α-CXCR3 was injected on day five post-therapy for the cohort which received two treatment cycles. Of note, a third treatment cycle was applied on day 13 post-inoculation (i.e. during the circulation time of the radiotracer) to ensure therapy effectiveness and sustained T cell activation over this period (see Suppl. Figure S7). Since the absolute organ uptakes both in reference organs such as blood or liver and in the target organs (tumor, spleen, lymph nodes) were found to vary between mice within the respective cohorts, uptake ratios (spleen/liver, spleen/blood, tumor/liver, tumor/blood) were calculated for intra-individual normalization and better comparability. Interestingly, the spleen-to-liver ratio (SLR) increased steadily from 1.08 ± 0.19 (untreated cohort) to 1.12 ± 0.09 (1xICI), and 1.54 ± 0.14 (3xICI) during treatment and was the lowest in the blocking cohorts (0.66 ± 0.083 control blocking and 0.65 ± 0.15 3xICI blocking), demonstrating CXCR3-specificity of [^64^Cu]Cu-NOTA-α-CXCR3 uptake in these tissues (Fig. 3d). Although differences between spleen-to-blood ratios (SBRs) were less significant, they showed identical trends (0.30 ± 0.04 (control), 0.39 ± 0.04 (1xICI), and 0.42 ± 0.07 (3xICI) vs. 0.17 ± 0.04 (3xICI blocking), see Fig. 3d). Furthermore, lymph-node-to-liver ratios followed the same pattern (from 0.42 ± 0.20 (control) to 0.77 ± 0.47 (1xICI) to 1.13 ± 0.35 (3xICI) vs. 0.17 ± 0.04 (3xICI blocking), see Suppl. Figure S8). As opposed to the secondary lymphoid organs, however, this treatment-dependent increase in tracer uptake was not observed for the MC38 tumors. Although tumor-to-liver ratios were marginally higher in treated mice, this increase was not significant.

### In vivo [^64^Cu]Cu-NOTA-α-CXCR3 PET/CT imaging in MC38 bearing mice

**Fig. 4:**
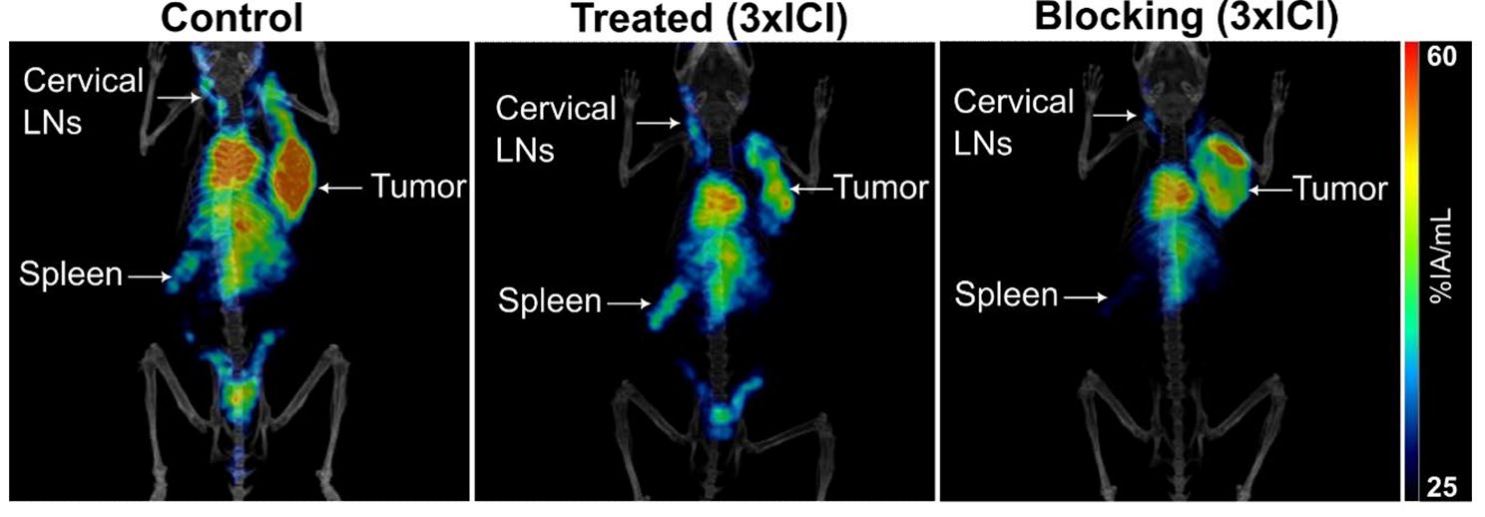
Representative [^64^Cu]Cu-NOTA- α-CXCR3 PET/CT images (maximum intensity projections, MIP) of mice bearing MC38 tumors on the right flank at 24 h p.i. Images show an untreated control mouse (left), a mouse having received 3 cycles of ICI (middle) and a mouse which received 3 cycles of ICI, coinjected with an 100-fold excess of α-CXCR3 mAb (blocking, right)

To validate the findings from the biodistribution studies and to investigate the sensitivity of [^64^Cu]Cu-NOTA-α-CXCR3 for detecting the observed ICI treatment effects in the secondary lymphoid organs, small animal [^64^Cu]Cu-NOTA-α-CXCR3 PET/CT imaging at 24 h p.i. was also performed (Fig. 4). Uptake values (VOI analysis) in the spleen were 28.9 (control), 26.3 (1xICI), 30.1 (3xICI) vs. 22.1%IA/g (3xICI blocking), respectively. Tracer uptake in the liver was 45.0 (control), 35.0 (1xICI), 36.8 (3xICI), and 39.3%IA/g (3xICI blocking), respectively. SLRs calculated from the representative PET/CT scans confirmed the results from the biodistribution experiment (i.e. an increase in the SLR from 0.64 (control) to 0.75 (1xICI treated) to 0.82 (3xICI treated) vs. 0.56 (3xICI blocking), see Suppl Figure S8). Figure 4 summarizes the imaging results obtained for representative mice (*n* = 1 per condition) taken from the respective cohorts that were used for the biodistribution study at 48 h p.i. (see previous section).

## Discussion

It is well established in the literature [15, 25] that T cell function as well as infiltration into the tumor is strongly dependent on CXCR3. High CXCR3 expression on activated T cells has not only been shown to be a robust predictor for the success of cell-based immunotherapies [19] but may also be upregulated in tumor-resident T cells due to immune checkpoint inhibition-induced T cell activation [15, 19]. To perform CXCR3-directed imaging, a mouse-specific α-CXCR3 antibody was conjugated with NOTA. Given that random conjugation of multiple macrocyclic chelators can influence the pharmacokinetics of an antibody [26], an established regioselective conjugation of NOTA to the Fc region was used to obtain the radiotracer precursor NOTA-a-CXCR3 [22]. With a DoC of 1.5 (NOTA chelators per antibody), negligible effects on the pharmacokinetics of the radiotracer compared to the unconjugated α-CXCR3 antibody were anticipated. Due to the site-specific chelator conjugation to the Fc region, effects on antibody affinity could also be safely excluded. The conserved affinity of the radiotracer was confirmed via a saturation binding assay, demonstrating specific binding to murine CXCR3 with nanomolar affinity.

The MC38 tumor model, along with other established models like CT26 and 4T1, belongs to highly immune infiltrated models and reveals notable responses to ICI therapy [27]. Thus, consistent with this finding, robust CXCR3^+^ cell infiltration was observed in the pilot experiments of this study, showing a progressive, ICI-induced increase in CXCR3^+^ T cell numbers and an improved infiltration of these cells in MC38 tumors by IF as well as by flow cytometry after two ICI treatment cycles. These data were also in accordance with results from CD3 immunohistochemistry of MC38 tumor slices, which in turn confirmed data from the literature [23]. An additional factor of high relevance for targeted imaging of immune-cell targets in the TME are the kinetics of T cell activation and expansion. In this study, flow cytometry analysis indicated that a single treatment cycle (three days post-treatment) did not yet have a detectable effect on activation and thus CXCR3 expression on tumor-infiltrating T cells but was only observed after 2 cycles of checkpoint inhibition five days post-treatment. This is a highly interesting finding and underlines the importance of characterizing the kinetics of the up- (and down)regulation of individual T cell activation markers in response to therapy. For example, another T cell activation marker, OX40 [28], was found do display very distinct kinetics compared to CXCR3. For OX40, imaging of early response at two days post-treatment was feasible, whereas at nine days post-treatment, the signal returned to baseline level.

Motivated by the sufficient baseline infiltration and increasing number of CXCR3^+^ T cells in the TME observed for the MC38 tumors under ICI therapy, the major aim of this study was to validate CXCR3 as an imaging target for T cell activation in the TME using the experimental radiotracer [^64^Cu]Cu-NOTA-α-CXCR3 as a robust, practicable, albeit not fully optimized tool.

Being the largest lymphoid organ, the spleen inherently showed a comparably high density of CXCR3^+^ cells in the initial IF experiments (Fig. 1D). Consequently, during the subsequent in vivo evaluation of [^64^Cu]Cu-NOTA-α-CXCR3, the spleen was considered as a positive control to verify the specificity of the radiotracer. Interestingly, even in this positive control tissue, low molar activities of the injected [^64^Cu]Cu-NOTA-α-CXCR3 and thus the comparably high amount of coinjected, unlabeled antibody (15 µg) precluded CXCR3-specific tracer accumulation (Suppl. Figure S6). Apparently, at this antibody dose, available CXCR3 receptors on splenic lymphoid T cells are already saturated by the excess of unlabeled antibody present in the radiopharmaceutical preparation. This observation is fully in line with previous results, where imaging of PD-1 expression was only feasible when small doses (2 µg) of the respective ^64^Cu-labeled PD-1 targeted antibody were used [29]. To circumvent these drawbacks, the following adjustments were implemented: [1] A 10-fold reduction of the injected dose to 1.5 µg per mouse, based on an optimized molar activity of [^64^Cu]Cu-NOTA-α-CXCR3 (300 GBq/µmol vs. 10 GBq/µmol), and [2] a later time point for the ex vivo biodistribution study (48 h p.i.) to allow more efficient tracer clearance and CXCR3-mediated accumulation. These adjustments resulted in specific and therapy-dependent radiotracer uptake observed in the spleen and lymph nodes (Fig. 3c-d and Table S1), for both of which high levels of CXCR3 expression was observed by IF.

Despite significant therapy-induced changes in specific uptake of [^64^Cu]Cu-NOTA-α-CXCR3 in the spleen and lymph nodes, limitations were encountered with respect to imaging changes in CXCR3^+^ T cell infiltration or CXCR3 upregulation on tumor-resident T cells. Under the chosen experimental conditions, no significant therapy-related change in tumor uptake of [^64^Cu]Cu-NOTA-α-CXCR3 was observed. We propose two possible explanations for this observation. First, the relatively low quantity of CXCR3^+^ expressing cells in the TME, even after 2 cycles of ICI treatment, limits the maximal achievable specific signal. For example, in the MC38 tumor model used in this study, a 6.7-fold increase in CXCR3^+^ T cell density was observed after ICI therapy. However, the cellular densities determined in the flow cytometry experiment ranged from 1524 *±* 579 CXCR3 + T cells/tumor (untreated control) to 9979 *±* 6369 cells/tumor (2xICI treated). This low number of target-expressing cells as well as a probably low expression level per cell are insufficient to obtain a CXCR3-specific signal using [^64^Cu]Cu-NOTA-α-CXCR3. With its affinity in the low nanomolar region (K_D_ = 3.3 nM), [^64^Cu]Cu-NOTA-α-CXCR3 aligns well with other radiolabeled antibodies used for imaging [30], but this affinity is not sufficient to sensitively detect low-level CXCR3 expression in the TME. Here, dramatically enhanced CXCR3-affinity (ideally in the pM region) would be required to obtain the desired sensitive signal such as for radiotracers based on e.g. pembrolizumab [31]. Second, the inherent pharmacokinetic properties of the full-sized antibody radiotracer, particularly its slow blood clearance, contribute to high non-specific background uptake in tumor tissue. This is further enhanced by the EPR effect and Fc-glycan-mediated uptake, both of which are well documented for antibody-based tracers [31, 32], favoring significant non-specific accumulation of the [^64^Cu]Cu-NOTA-α-CXCR3 macromolecule in the tumor tissue. As mentioned, MC38 tumors are highly immune infiltrated, and additional effects such as the efficient capture of the radiolabeled antibody by tumor associated macrophages via Fcg receptors and its subsequent retention in this cell population is also a possible mechanism contributing to the high CXCR3 independent tumor uptake of [^64^Cu]Cu-NOTA-α-CXCR3 [33].

All these combined effects may contribute to the high absolute tumor uptake of [^64^Cu]Cu-NOTA-α-CXCR3 and thus mask small, but significant differences of in CXCR3-mediated radiotracer uptake by CXCR3-positive T cells in the TME. On the other hand, as an inverse effect, efficient extravasation of macromolecules such as full-size antibodies into the tumor tissue is limited [34], and thus, [^64^Cu]Cu-NOTA-α-CXCR3 may be unable to reach the T cells residing in the TME within the observed time window. As a consequence, the utility of the present antibody-based approach for imaging activation of small T cell (sub)populations in the TME via CXCR3 targeting is very limited. As mentioned, next generation CXCR3-targeted radiotracers with extremely high affinities (pM), and optimized, adapted pharmacokinetics and tissue penetration may have the potential to solve these issues.

However, despite its limitations in TME imaging, [^64^Cu]Cu-NOTA-α-CXCR3 allowed in vivo imaging of systemic T cell activation, as demonstrated by an increased tracer uptake in the spleen and in the lymph nodes (Fig. 3) upon ICI treatment. Thus, the present antibody-based approach may at least serve as a broader strategy [35] to image T cell activation. High splenic activity was recently proposed as a positive indicator of successful immunotherapy in melanoma and Hodgkin lymphoma patients using 2-[^18^F]FDG [36, 37]. Furthermore, previous studies have shown that the T cell activation marker CD69 exhibits increased expression in spleens of mice responding to ICI, whereas non-responders and untreated mice showed lower splenic uptake of the CD69-targeted [^89^Zr]-DFO-H1.2F3 [38]. This suggests that, triggered by ICI, the number of effector T cells in the lymphatic circulation increases and immunological remodeling is taking place in the spleen [39]. As shown for the spleen-to-liver and spleen-to-blood ratios used in this study (Fig. 3d), systemic response imaging could thus be feasible using [^64^Cu]Cu-NOTA-α-CXCR3 PET. However, this assumption requires thorough further investigation since in the present study, animal cohorts were not sufficiently large to differentiate between ICI therapy responders and non-responders. Only if a correlation between the spleen-to-liver and spleen-to-blood ratios of [^64^Cu]Cu-NOTA-α-CXCR3 uptake of responders and non-responders can be observed, [^64^Cu]Cu-NOTA-α-CXCR3-based PET imaging may qualify as an imaging approach for assessing systemic T cell activation as a surrogate marker for tumor response to ICI therapy. Of note, the applicability of CXCR3 imaging is not limited to immunotherapy response imaging. Similar to CXCR4-directed imaging [40], this receptor can also be exploited as a biomarker for accurately monitoring acute inflammation [20, 21]. Therefore, additional studies in the broader context of T cell activation imaging will certainly provide interesting insights.

## Conclusions

In summary, the results of this study suggest that in principle, CXCR3 represents a relevant molecular marker for T cell activation in an immunotherapy setting. The antibody-based imaging approach using [^64^Cu]Cu-NOTA-α-CXCR3 was able to unambiguously demonstrate systemic T cell activation, as reflected by tracer uptake in CXCR3^+^ T cell rich organs such as spleen and lymph nodes. To be able to further exploit this highly interesting molecular marker as a target for imaging T cell activation in the TME, however, an optimized tracer design is required, providing not only very high affinity to CXCR3, ideally in the pM range, but also reduced background accumulation and faster general accumulation and clearance kinetics. First attempts in this direction have been reported [21] but require further optimization. Overall, this study represents an important first step towards establishing CXCR3 as an important (early) T cell activation marker alongside CD69, OX40, ICOS, IL-2R, IFN-γ, Granzyme B as well as T-cell metabolism and in the future may attract considerable clinical interest, as soon as an optimized tracer concept is available.

### Electronic supplementary material

Below is the link to the electronic supplementary material.

Supplementary Material 1

## Data Availability

All data generated or analysed during this study are included in this published article [and its supplementary information files].

## Abbreviations

CXCR3: C-X-C chemokine receptor 3
TME: tumor micro-environment
ICI: immune checkpoint inhibitor
NOTA: 2,2,2-(1,4,7-triazacyclononane-1,4,7-triyl)triacetic acid
TIL: tumor infiltrating lymphocyte
CAR-T: chimeric antigen receptor T cell
PET: positron emission tomography
SPECT: single photon emission computed tomography
ICOS: inducible T cell COStimulator
IL-2R: interleukin-2 receptor
CXCL9: C-X-C chemokine ligand 9
PD-1: programmed cell death protein 1
CTLA-4: cytotoxic T-lymphocyte-associated protein 4
mAb: monoclonal antibody
DoC: degree of conjugation
EDTA: ethylenediaminetetraacetic acid
SEC: size exclusion chromatography
RCP: radiochemical purity
iTLC: instant thin layer chromatography
RCY: radiochemical yield
BSA: bovine serum albumine
FBS: fetal bovine serum
IHC: immunohisotchemistry
IF: immunofluorescence
MA: molar activity
K_D_: dissociation constant
SLR: spleen-to-liver ratio
SBR: spleen-to-blood ratio
