# Supplementary material for: Validation of the C-X-C chemokine receptor 3 (CXCR3) as a target for PET imaging of T cell activation
Source: EJNMMI Res. 2024 Aug 28;14:77. doi: 10.1186/s13550-024-01142-1 (PMC11358572; doi:10.1186/s13550-024-01142-1)
Supplement: Supplementary file 1 — Supplementary Material 1 [file 13550_2024_1142_MOESM1_ESM.docx]

Electronic Supplementary Material

Validation of the C-X-C chemokine receptor 3 (CXCR3) as a target for PET imaging of T cell activation

Sebastian Martin^1,2,3^, Lennard Wendlinger^1,2,3^, Béatrice Zitti^2,5^, Mehdi Hicham^2,5^, Viktoriia Postupalenko^4^, Léo Marx^4^, Greta Giordano-Attianese^6^, Elisabetta Cribioli^6^, Melita Irving^6^, Alexandra Litvinenko^1,2,3^, Radmila Faizova^1,2,3^, David Viertl^1,2,3^, Margret Schottelius^1,2,3^

*^1^ Translational Radiopharmaceutical Sciences, Department of Nuclear Medicine and Department of Oncology, Centre Hospitalier Universitaire Vaudois (CHUV) and University of Lausanne (UNIL), 1011 Lausanne, Switzerland*

*^2^ AGORA, Pôle de recherche sur le cancer, 1011 Lausanne, Switzerland*

*^3^ SCCL Swiss Cancer Center Leman, 1011 Lausanne, Switzerland*

*^4^ Debiopharm Research & Manufacturing SA, Campus “après-demain”, Rue du Levant 146, 1920 Martigny, Switzerland*

*^5^ Department of Pathology and Immunology, University of Geneva, Geneva, Switzerland*

*^6^ Ludwig Institute for Cancer Research, University of Lausanne, 1066 Epalinges, Switzerland; Department of Oncology, University Hospital of Lausanne, 1011 Lausanne, Switzerland*

***Corresponding Author:**

Prof. Dr. Margret Schottelius

Phone +41.21.545.1120

Mobile +41.79.556.0143

Email: margret.schottelius@chuv.ch

*Protein Mass Spectrometry*

The mass spectrometry was performed on a Q ExactiveTM HF Orbitrap operating in the protein mode in mass ranges of 15 kDa (EPFL, Sion). The scans were set in the range of 1200-4000 m/z at a resolution of 15 k and a SID of 25 eV. The mass spectra were deconvolved using Protein Deconvolution 4.0 (Thermo Scientific). For the measurement the samples were passed through a Acquity UPLC Protein BEH C4 column (1x150mm, 1.7 µm, 300 Å, Waters) at a flow rate of 90 µL/min. The column oven was set at 60°C. Gradient: from 25% to 40% within 12 min. Solvent A: water +0.1% formic acid, solvent B: acetonitrile + 0.1% formic acid. Before the injection of 6 µL sample volume the samples were diluted 100 times with solvent A.

*Cell transfection*

For the retroviral harvest 293T cells were used as packaging cell line. 8x10^6^ cells were seeded in a T150 flask the day before of the transfection in 16.5 mL of complete RPMI media. The cells were transfected by using Turbofect with 42 µg of total DNA (gagpol 14 µg, transfer vector 21 µg, envelop 7 µg). Turbofect and Optimem have been used according to the manufacturer’s instructions. 48 hours after the transfection the supernatant was collected and concentrated by a ultracentrifugation at 24x105xg for 1.5 h. Next, 300x10^5^ CHO cells were infected with 100 µL of concentrated virus. Phenotype control of the target protein was performed 5 days after transduction by flow cytometry. The cells were maintained and expanded in DMEM/F12 containing 10% FBS and 1% Penicillin/Streptomycin.

*Flow cytometry of in vitro cultivated cells*

CHO-CXC3 or MC38 cells were detached using 2 mM EDTA in PBS for 15-20 min at 37°C. The cells were washed twice with cold flow cytometry buffer (1% fetal bovine serum in PBS). For immunofluorescence staining, 1x10^6^ cells were prepared and incubated for 30 min on ice with 100 µL flow cytometry buffer contianing 1 μg/mL of PE anti-mouse CXCR3 (BioLegend, #Cat: 126505, Clone: CXCR3-173). An unstained sample was used as negative control. The cells were washed two times with 200 μL cold flow cytometry buffer. DAPI was added to each sample shortly before the analysis to yield a final concentration of 0.5 µg/mL. The flow cytometry analyses were conducted on the same day on a Beckman Coulter Gallios flow cytometer. The acquired data were analyzed with FlowJo v10.7.1.

**Table S 1**: Biodistribution of [^64^Cu]Cu-NOTA-α-CXCR3 in MC38 tumor bearing C57Bl/6 mice at 48 hours p.i.. Uptake values are expressed as %IA/g (%-injected activity per gram of tissue) and are means ± SD (n=5 mice per group). Groups include untreated mice (Control), mice treated with 1 cycle of ICI (1xICI), mice treated using 3 cycles of ICI (3xICI). Blocking of 3xICI treated and blocking of untreated control mice was conducted using a 100-fold molar excess of α-CXCR3 antibody.

| **Organ** | **Control** | **Blocking (untreated control)** | **1xICI** | **3xICI** | **Blocking (3xICI)** |
| --- | --- | --- | --- | --- | --- |
| Blood | 19.3±2.1 | 19.96±2.36 | 16.69±1.37 | 16.30±4.29 | 20.10±1.56 |
| Heart | 4.62±0.45 | 5.31±1.64 | 4.62±0.95 | 5.14±1.86 | 6.27±1.03 |
| Lungs | 7.30±0.94 | 8.91±2.61 | 6.10±0.63 | 6.85±3.58 | 7.21±0.53 |
| Liver | 5.62±0.39 | 5.85±1.30 | 5.79±0.28 | 4.46±1.44 | 5.34±0.41 |
| Pancreas | 1.85±0.14 | 1.82±0.47 | 1.69±0.22 | 2.59±0.57 | 2.55±0.91 |
| Spleen | 6.04±1.02 | 3.84±0.79 | 6.48±0.45 | 6.19±2.11 | 3.51±0.99 |
| Kidneys | 5.22±0.26 | 5.65±1.08 | 5.45±0.46 | 5.01±1.35 | 6.08±0.80 |
| Intestines | 2.09±0.36 | 1.54±0.78 | 2.06±0.25 | 1.78±0.46 | 1.99±0.22 |
| Stomach | 1.97±0.16 | 1.92±0.35 | 1.69±0.38 | 1.82±0.68 | 1.95±0.23 |
| Bone | 2.38±0.33 | 1.83±0.54 | 2.14±0.38 | 1.91±0.78 | 2.08±0.08 |
| Muscle | 0.89±0.08 | 1.16±0.18 | 0.90±0.14 | 1.09±0.20 | 1.10±0.38 |
| Tumor | 11.45±1.77 | 10.67±2.85 | 13.86±3.10 | 9.42±3.68 | 10.85±2.39 |
| Lymph nodes | 6.33±1.25 | 3.95±0.73 | 5.50±1.07 | 4.78±1.40 | 2.51±1.23 |


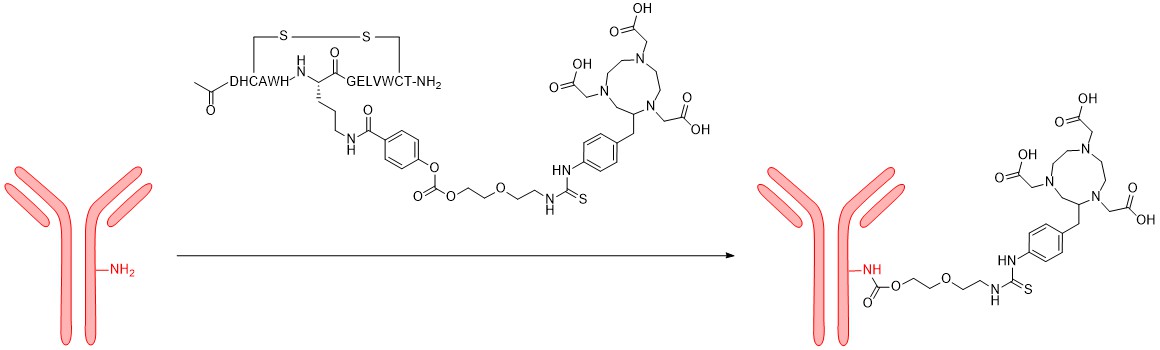


**Figure S1**: AbYlink^TM^ technology for Fc-site specific antibody conjugation. Patent application WO 2022/079031.


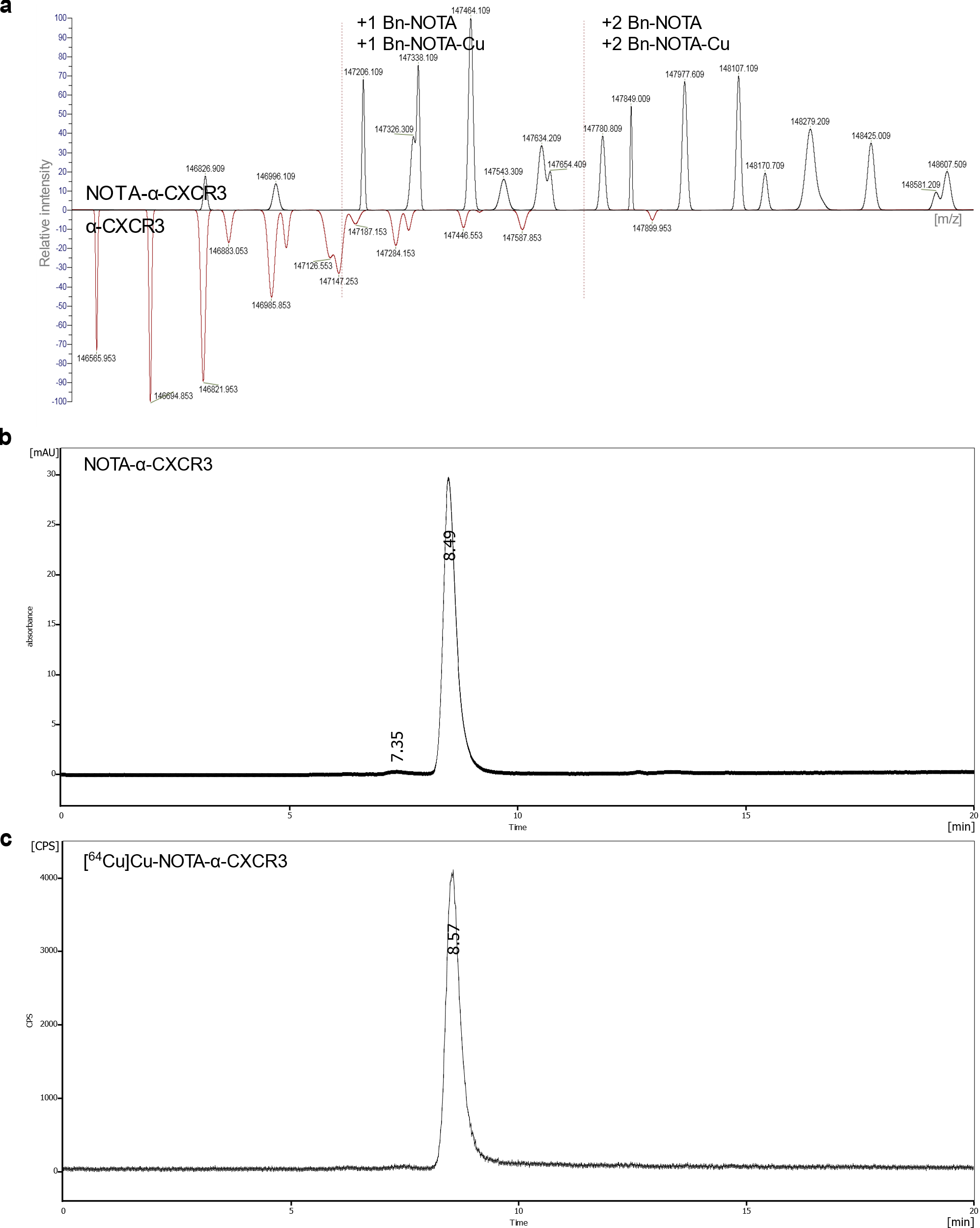


**Figure S2**: (**a**) Evaluation of the degree of conjugation (DoC) through ESI mass

spectrometry. The NOTA-conjugated antibody is illustrated on the top and on the bottom the unconjugated antibody as control. Bn-NOTA: +582 m/z, and BN-NOTA-Cu: +643 m/z. (**b**) SEC-chromatogram of NOTA-α-CXCR3 in labeling buffer (0.1 M NaOAc, pH 5.5), R_t_ 8.49 min; (**c**) Radio-SEC chromatogram of [^64^Cu]Cu-NOTA-α-CXCR3, R_t_ 8.57 min. The SEC was performed using a XBridge protein BEH 200A SEC 3.5 µm, 7.8 x 300 mm (Waters,Baden-Dättwil, Switzerland) and phosphate buffer (pH = 6.8, 0.1 M) containing 342 mM NaCl as mobile phase at a constant flow of 1 mL/min.


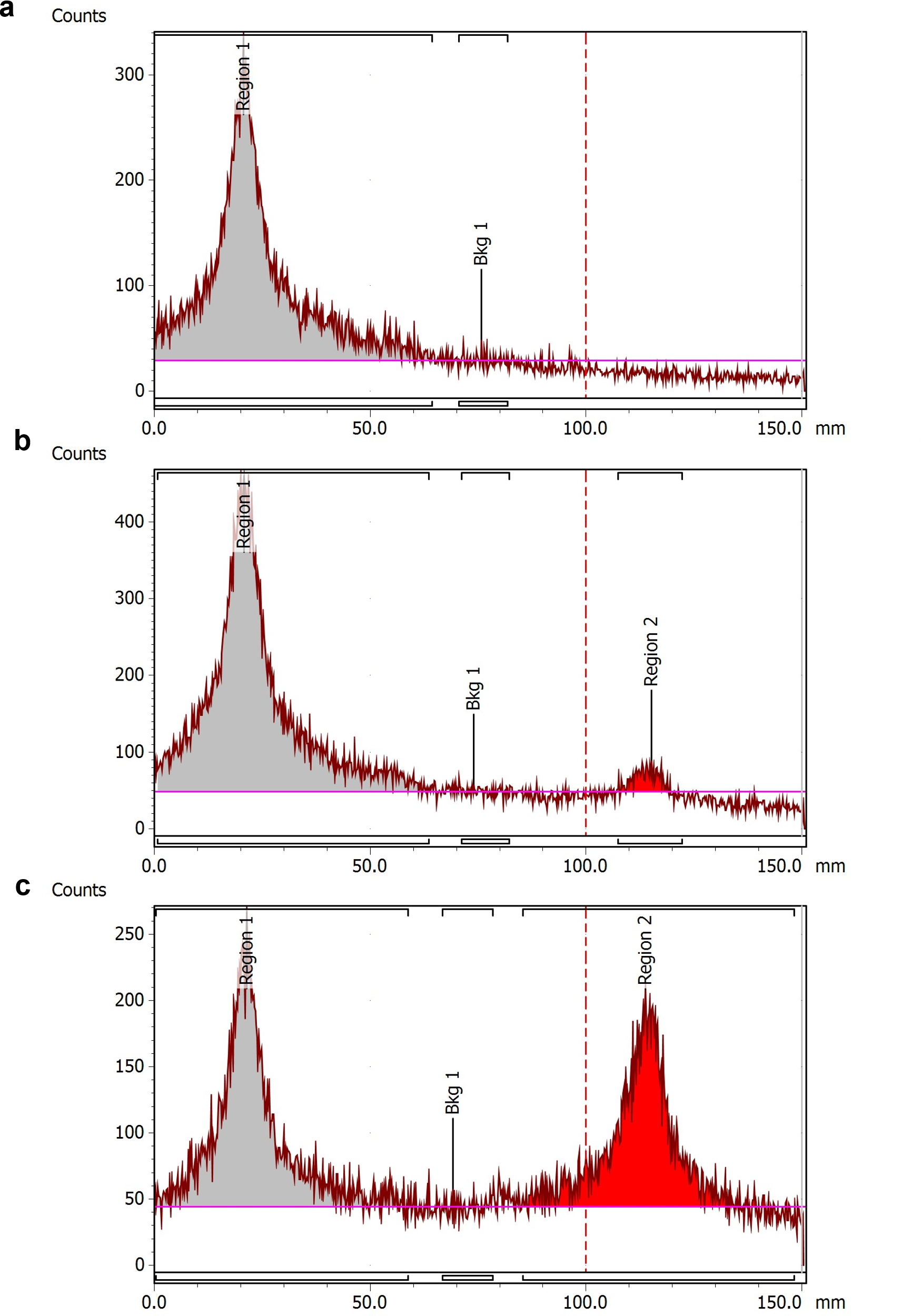


**Figure S3**: ITLC chromatograms of [^64^Cu]Cu-NOTA-α-CXCR3 radiolabeled with increasing activities of ^64^Cu. Molar activity (MA) was calculated by dividing the decay-corrected activity at the start of synthesis by the molar mass of the full-size antibody (150 kDa). Region 1: [^64^Cu]Cu-NOTA-α-CXCR3; Region 2: [^64^Cu]Cu-EDTA; Bkg1: Background region. (**a**) Radiolabeling to MA of 538 GBq/µmol; (**b**) MA of 690 GBq/µmol; (**c**) MA of 1250 GBq/µmol.
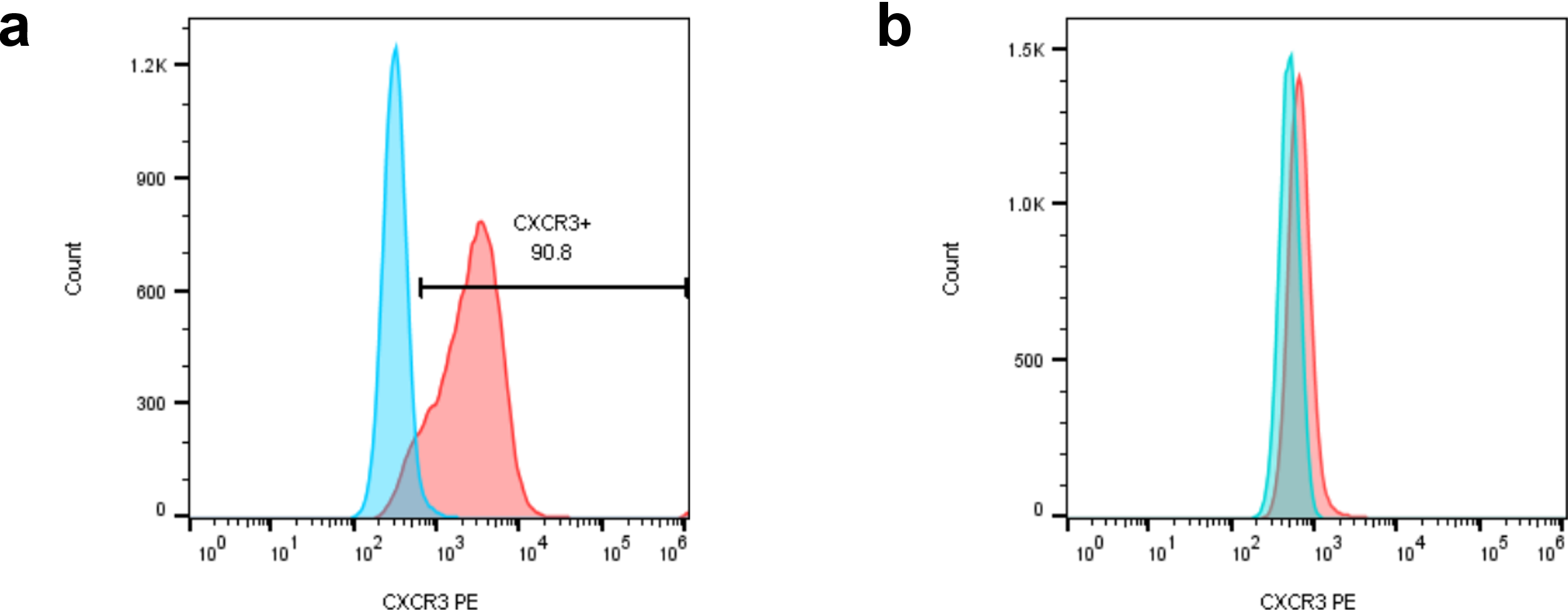


**Figure S4**: Flow cytometry analysis. Anti-mouse α-CXCR3-PE stained cells in red, unstained cells in blue. (**a**) Transfected CHO cells expressing murine CXCR3; (**b**) MC38 tumor cells.


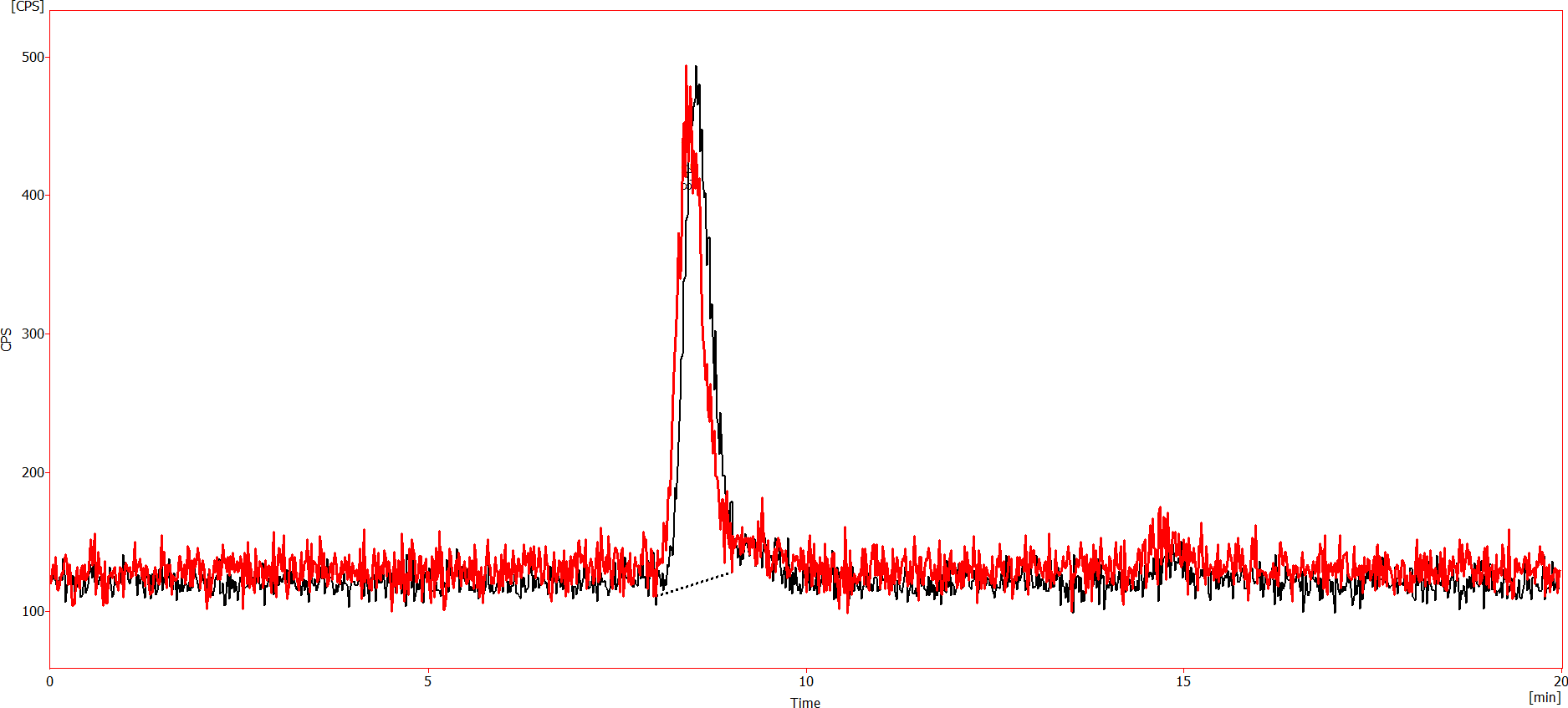


**Figure S5**: Stability of [^64^Cu]Cu-NOTA-α-CXCR3 in human serum. Overlay of SEC analyses after 24 hours (red) and 48 hours (black) of incubation at 37°C.


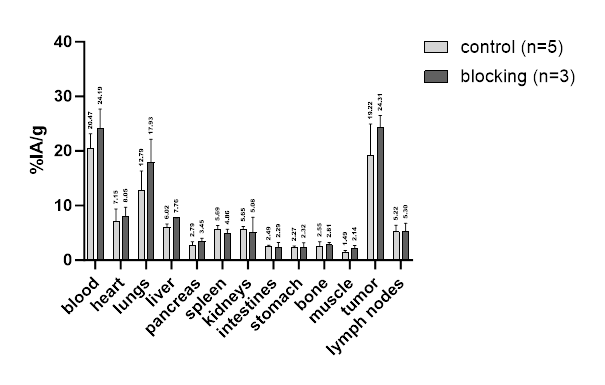


**Figure S6**:  Biodistribution of [^64^Cu]Cu-NOTA-α-CXCR3 (10 GBq/µmol, 15 µg mAb/mouse) at 24h p.i. in untreated MC38 tumor bearing mice.


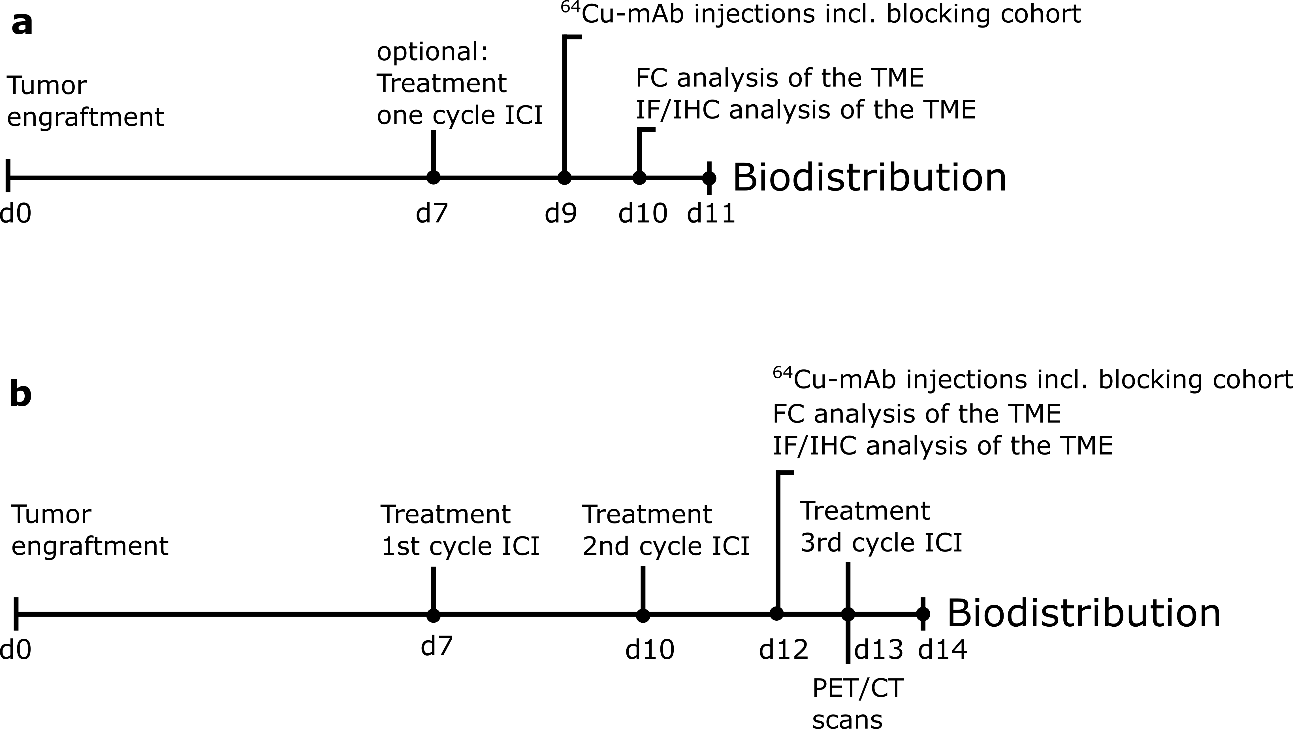


**Figure S7**: ICI treatment schedule for MC38 tumor bearing mice. ICI: immune checkpoint inhibitor, IF: immunofluorescence, mAb: monoclonal antibody. (a) Schedule for untreated mice and mice treated with one dose of ICI. (b) Schedule for mice which received two or three doses of ICI.


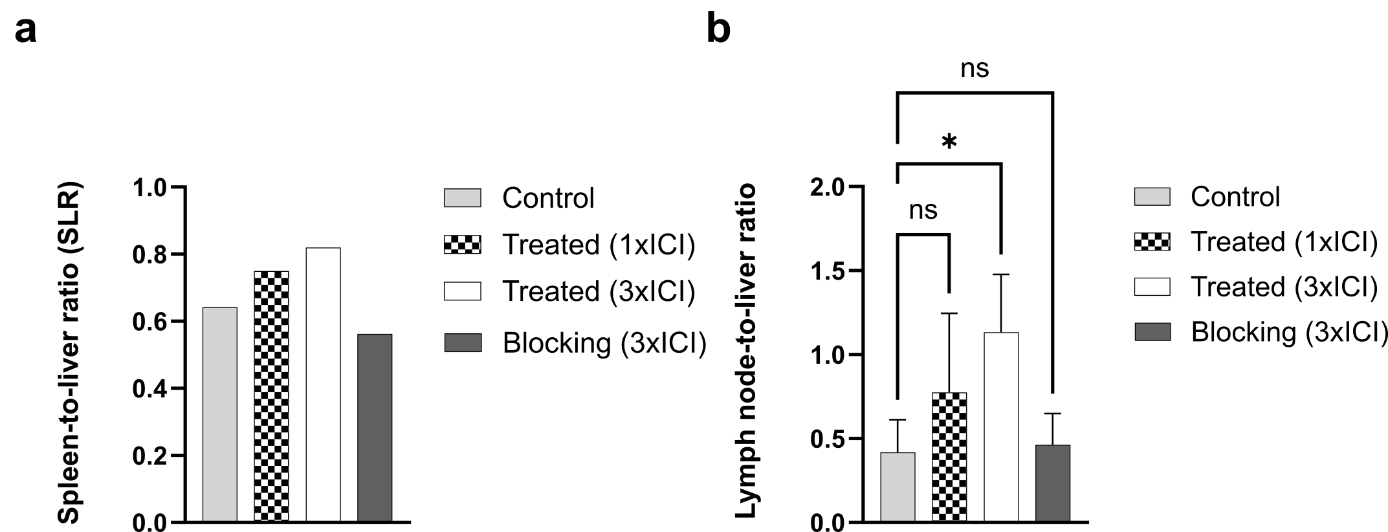


**Figure S8**: (**a**) Spleen-to-liver ratios as determined from VOI evaluation of the representative PET/CT images (n=1 each condition); (**b**) Lymph node-to-liver ratios as determined from the Biodistribution data (**Table S1**).


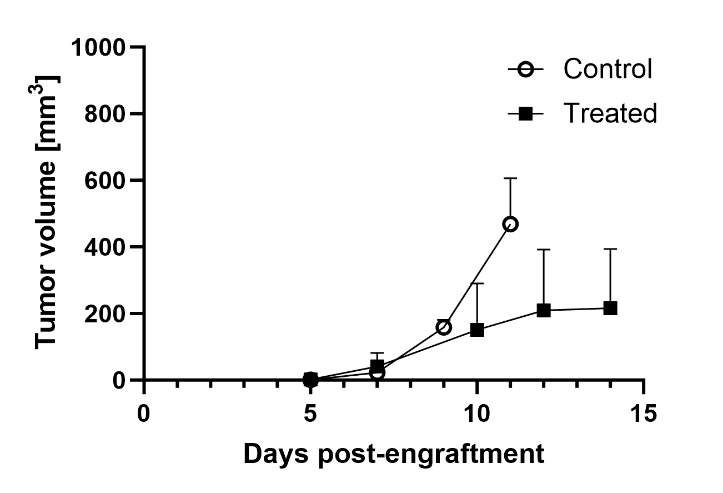


**Figure S9**: Growth kinetics of MC38 tumors in CB57Bl/6 mice. Tumors (2x10^6^ cells) were engrafted subcutaneously on the right flank. Immune checkpoint inhibitor treatment using αPD1 and αCTLA-4 was applied on day 7, 10 and 13 post-engraftment.
